# Description of Two Resistance-Nodulation-Cell Division Efflux Systems Involved in Acquired Antibiotic Resistance: AxySUV in *Achromobacter xylosoxidans* and AinCDJ in *Achromobacter insuavis*

**DOI:** 10.3390/antibiotics14060536

**Published:** 2025-05-23

**Authors:** Arnaud Magallon, Julien Bador, Thomas Garrigos, Caroline Demeule, Anaïs Chapelle, Véronique Varin, Catherine Neuwirth, Lucie Amoureux

**Affiliations:** 1 Department of Bacteriology, University Hospital of Dijon, BP 37013, 21070 Dijon Cedex, France; julien.bador@chu-dijon.fr (J.B.); caroline.demeule@chu-dijon.fr (C.D.); anaischapelle21600@gmail.com (A.C.); veronique.varin@chu-dijon.fr (V.V.); catherine.neuwirth@chu-dijon.fr (C.N.); lucie.amoureux@chu-dijon.fr (L.A.); 2 UMR AgroEcologie 1347, INRAE, University of Bourgogne, 21065 Dijon Cedex, France; 3 Department of Bacteriology, University Hospital of Félix Guyon, 97400 Saint-Denis, France; thomas.garrigos@chu-reunion.fr

**Keywords:** *Achromobacter*, *A. xylosoxidans*, *A. insuavis*, antibiotic resistance, efflux, RND pumps, AxySUV, AinCDJ, fluoroquinolones, carbapenems

## Abstract

Background/Objectives: *Achromobacter xylosoxidans* and *Achromobacter insuavis* are emerging opportunistic pathogens. Several Resistance-Nodulation-cell Division (RND) efflux systems are involved in intrinsic or acquired antibiotic resistance (AxyABM, AxyXY-OprZ, and AxyEF-OprN). The aim of this study was to explore the resistance mechanisms in one-step mutants in which the efflux systems described to date are not involved: one mutant of *A. insuavis* AXX-A (AXX-A-Do1) and two mutants of *A. xylosoxidans* CIP102236 (CIP102236-El9 and CIP102236-Eo4) selected on fluoroquinolones. Methods: In vitro mutants were compared to parental isolates by WGS. RT–qPCR and gene inactivation were used to explore the role of the new efflux systems detected. Results: In the *A. insuavis* AXX-A mutant (AXX-A-Do1), WGS showed a substitution in the putative regulator of the new RND efflux system AinCDJ. The transporter gene *ainD* was 79-fold overexpressed in AXX-A-Do1, compared to its parental strain. The inactivation of *ainD* in AXX-A-Do1 led to a decrease in MICs of ciprofloxacin (8-fold), levofloxacin (8-fold), cefepime (≥8-fold), meropenem (4-fold), doripenem (4-fold), doxycycline (4-fold), minocycline (4-fold), tigecycline (4-fold) and chloramphenicol (≥8-fold). The MICs values obtained were similar to those of the parental strain AXX-A. The same approach allowed the detection of the new efflux system AxySUV in *A. xylosoxidans* CIP102236 mutants, in which substitutions in the putative AxySUV regulator were associated with the overexpression of the transporter gene *axyU*. *axyU* inactivation in the mutants led to a decrease in MICs of ciprofloxacin (8- to 16-fold), levofloxacin (4- to 8-fold), doripenem (4-fold), doxycycline (4-fold), minocycline (4-fold), and chloramphenicol (≥4-fold). Interestingly, *axySUV* is present in only about 50% of available *A. xylosoxidans* genomes, whereas *ainCDJ* is detected in all *A. insuavis* genomes. Conclusions: This study demonstrated that AinCDJ overproduction is involved in the acquired resistance of *A. insuavis* to cefepime, meropenem, doripenem, fluoroquinolones, minocycline, doxycycline, tigecycline, and chloramphenicol and that AxySUV overproduction is involved in the acquired resistance of *A. xylosoxidans* to meropenem, fluoroquinolones, minocycline, doxycycline, and chloramphenicol.

## 1. Introduction

*Achromobacter* are non-fermentative Gram-negative bacilli considered as emerging opportunistic pathogens, mainly among patients with cystic fibrosis (CF), immunocompromised patients, or patients with healthcare-associated infections [1]. Accurate species identification requires the sequencing of the housekeeping gene *nrdA*, MultiLocus Sequencing Typing (MLST) analysis, or the use of MALDI-TOF MS with an adequate database [2]. The most common species found in clinical samples is *Achromobacter xylosoxidans* (*A. xylosoxidans*), followed by *Achromobacter insuavis* (*A. insuavis*) or *Achromobacter ruhlandii* (*A. ruhlandii*), depending on the country [3,4,5]. Intrinsic antibiotic resistance, combined with frequent acquired resistance, often makes treatment complex [2]. The understanding of resistance mechanisms in *Achromobacter* is still limited, but the contribution of three Resistance-Nodulation-cell Division (RND) efflux systems, AxyABM, AxyXY-OprZ, and AxyEF-OprN [6,7,8,9,10,11,12], has been clearly demonstrated. RND-type efflux systems are tripartite and consist of a membrane fusion protein (MFP), an RND transporter, and an outer membrane factor (OMF), encoded by three genes [13]. These three proteins form a channel enabling the extrusion of various drugs, including antibiotics, into the extracellular environment. AxyABM is involved in the low natural susceptibility to cefotaxime, temocillin, and aztreonam [7,10]. AxyXY-OprZ is responsible for the natural resistance of *A. xylosoxidans* and *A. insuavis* to aminoglycosides [8]. AxyEF-OprN is not involved in intrinsic resistance [6]. The overproduction of these systems, due to substitutions or deletions in their respective local regulators, leads to acquired resistance in *Achromobacter* [6,7,12,14,15]. Acquired resistance to fluoroquinolones (FQs) has been associated with the overexpression of AxyABM (combined with increased MICs of carbapenems, ceftazidime, cefepime, cyclines, cefiderocol, and trimethoprim/sulfamethoxazole) [7], AxyXY-OprZ (combined with increased MICs of aminoglycosides, cefepime, and tetracyclines) [12], or AxyEF-OprN [6,15]. In contrast with Enterobacterales and *Pseudomonas aeruginosa*, substitutions in *Achromobacter* quinolone-resistance-determining regions (QRDRs) are less frequently involved [6]. Nevertheless, in some clinical strains, FQ resistance is not linked to any of the mechanisms listed above.

In a previous work, in an attempt to highlight undescribed resistance mechanisms, we selected in vitro one-step FQ resistant mutants from the strains *A. xylosoxidans* CIP102236 and *A. insuavis* AXX-A [6]. Some of them harbored neither substitutions in QRDR nor the overexpression of any of the three efflux systems described above but, in addition to FQ resistance, exhibited higher doripenem, cyclines, and chloramphenicol MICs than their parental strains, suggesting the involvement of efflux. In the present study, we aimed to explore the resistance mechanisms involved in these mutants (two selected from *A. xylosoxidans* CIP102236—CIP102236-El9 and CIP102236-Eo4—and one from *A. insuavis* AXX-A—AXX-A-Do1).

## 2. Results

### 2.1. Description of AinCDJ in A. insuavis

**Figure 1:**
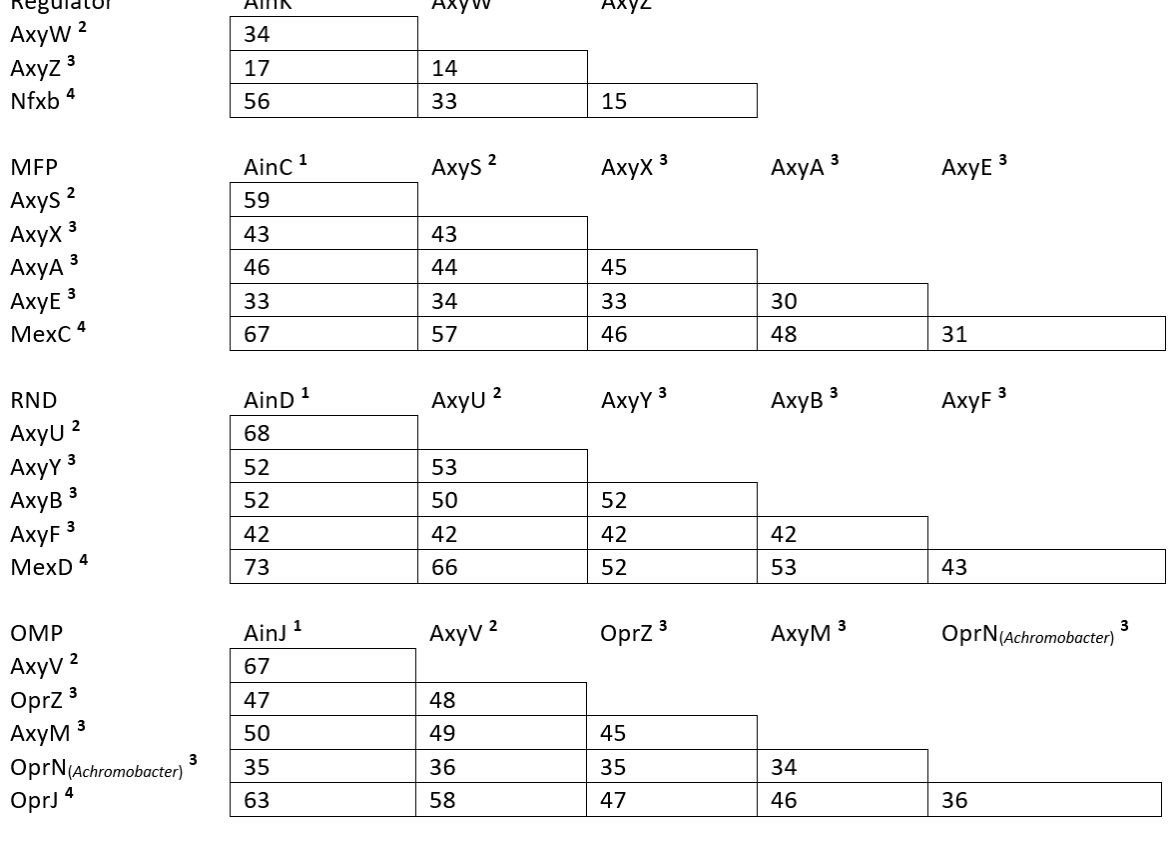
Amino acid sequence similarities (%) for each of the protein components (Regulator, MFP, RND and OMP) of the following efflux systems: AxySUV, AinCDJ, AxyABM, AxyXY-OprZ, AxyEF-OprN, and MexCD-OprJ. The protein sequences used for the comparisons were sourced from the following strains: ^1^ AXX-A, *A. insuavis* (Genbank accession numbers: EGP44028, EGP44029, EGP44030, and EGP44031); ^2^ CIP102236, *A. xylosoxidans* (GenBank accession numbers: OP186062, OP186063, OP186064, and OP186065); ^3^ ATCC27061, *A. xylosoxidans*^T^ (Genbank accession number: BCZG00000000.1); ^4^ PAO1, *P. aeruginosa* (Genbank accession numbers: NP_253290, NP_253289, NP_253288, and NP_253287).

The mutant AXX-A-Do1 harboring decreased susceptibility to FQ, cefepime, doripenem, cyclines, and chloramphenicol was compared to its parental strain, *A. insuavis* AXX-A, by means of WGS analysis. AXX-A-Do1 harbored the mutation T41A in a TetR-family transcriptional regulator (TFTR) gene, which was named *ainK* (accession number: EGP44028). This non-synonymous mutation in *ainK* was confirmed by Sanger sequencing and resulted in the amino acid substitution L14Q. Interestingly, this gene is located upstream of three genes encoding an uncharacterized RND-type efflux system (accession numbers: EGP44029, EGP44030, and EGP44031). Protein sequence alignment of the components of this system showed high protein sequence similarity with MexCD-OprJ from *P. aeruginosa PAO1:* 67% with MexC (NP_253289), 73% with MexD (NP_253288), and 63% with OprJ (NP_253287) (Figure 1 and Figures S1–S4). Therefore, we named this system AinCDJ: AinC (MFP), AinD (RND transporter), and AinJ (OMP). AinK shared 56% amino acid similarity with Nfxb, the regulator of MexCD-OprJ (NP_253290). Using the Operon_Mapper online tool (https://biocomputo.ibt.unam.mx/operon_mapper/), the three components (MFP, RND transporter, and OMF-encoding genes) were mapped as being associated within the same operon. The *ainK* regulator gene was the single gene of another operon. In silico modeling using the online tool Swiss-model (https://swissmodel.expasy.org/) indicated that the substitution L14Q is located in the helix 1 of the N-terminal DNA-binding domain of AinK (Figure S5).

In the mutant AXX-A-Do1, RT-qPCR revealed a 79-fold overexpression (mean value of the six measures) of *ainD* (encoding the transporter) compared to its parental strain AXX-A. The inactivation of *ainD* in AXX-A-Do1 resulted in a decrease in MICs for several antibiotics (to the level of AXX-A MICs): ofloxacin (≥16-fold), levofloxacin (8-fold), ciprofloxacin (8-fold), cefepime (≥8-fold), doripenem (4-fold), meropenem (4-fold), doxycycline (4-fold), minocycline (4-fold), tigecycline (4-fold), and chloramphenicol (≥8-fold) (Table 1). This result confirms the implication of AinCDJ in AXX-A-Do1 acquired resistance. In contrast, the inactivation of *ainD* in the parental strain AXX-A did not significantly modify the antibiotic MICs.

The regulator AinK could act as a repressor of AinCDJ because the inactivation of the corresponding gene *ainK* in AXX-A led to (i) a drastic MIC increase of the ten antibiotics listed above, and (ii) a 55-fold higher expression (mean value) of *ainD* compared with the parental strain AXX-A. Furthermore, the inactivation of *ainK* in AXX-A-Do1 did not significantly modify the antibiotic MICs, demonstrating that the substitution L14Q in AinK has similar effects to the *ainK* inactivation in this mutant.

Among the 597 available *Achromobacter* genomes in Genbank (https://www.ncbi.nlm.nih.gov/datasets/genome/?taxon=222, accessed on 26 March 2025), we identified 24 *A. insuavis* genomes. The system AinCDJ was detected in all *A. insuavis* genomes and was highly conserved (>92% nucleotidic similarity for the entire sequence from *ainK* to *ainJ*). No mutation leading to L14Q amino acid substitution in AinK has been detected in *A. insuavis* available genomes.

Concerning the genomic context of the AinCDJ system in AXX-A, the genes encoding AinCDJ are located at the same locus in all genomes, downstream of a gene encoding a putative oxidoreductase.

### 2.2. Description of AxySUV in A. xylosoxidans

The mutants CIP102236-Eo4 and CIP102236-El9 were compared to their parental strain CIP102236 by means of WGS analysis. CIP102236-Eo4 and CIP102236-El9 harbored non-synonymous mutations (C110T leading to A37V and T38A leading to L13Q, respectively, as confirmed by Sanger sequencing) in a TFTR gene located upstream of three genes encoding a novel uncharacterized RND-type efflux system. When compared to efflux systems of *P. aeruginosa* PAO1, the highest protein sequence similarity was observed with MexCD-OprJ but limited to 33% for the regulator, 57% for the MFP, 66% for the transporter, and 58% for the OMP (Figure 1). Protein sequence alignment of the components of this system with those of the system AinCDJ from *A. insuavis* showed the following similarities: 34% for the regulator, 59% for the MFP, 68% for the RND transporter, and 67% for the OMP (Figure 1 and Figures S1–S4). Because of the poor amino acid similarities with RND efflux systems described to date, we decided to name this system AxySUV: AxyS (MFP), AxyU (RND transporter), AxyV (OMP), and AxyW (TFTR). These sequences were deposited in Genbank. MFP, RND transporter, and OMF-encoding genes were indicated as being associated within the same operon by the Operon_Mapper online tool. The *axyW* regulator gene was the single gene of another operon.

The pump gene *axyU* was 138-fold and 37-fold (mean values) overexpressed in the mutants CIP102236-Eo4 and CIP102236-El9, respectively, compared to the parental strain CIP102236. The inactivation of *axyU* in the mutants CIP102236-Eo4 and CIP102236-El9 led to a decrease in MICs of ofloxacin (≥4 to ≥8-fold), levofloxacin (4 to 8-fold), ciprofloxacin (8 to 16-fold), doripenem (4-fold), doxycycline (4-fold), minocycline (4-fold), and chloramphenicol (≥4-fold) (Table 2). The MIC values obtained in CIP102236-Eo4-∆U and CIP102236-El9-∆U were similar to those of CIP102236. Conversely, the inactivation of *axyU* in the parental strain CIP102236 did not significantly modify the MICs of the antibiotics tested.

The inactivation of *axyW* in the parental strain CIP102236 led to a drastic increase in MICs of the antibiotics listed above, with MIC values close to those of the mutants CIP102236-Eo4 and CIP102236-El9 (Table 2). The pump gene *axyU* was 41-fold (mean value) overexpressed in CIP102236-∆W compared to the parental strain CIP102236. Therefore, AxyW seems to act as a repressor of AxySUV. The inactivation of *axyW* in the two mutants did not significantly increase the antibiotic MICs (Table 2), demonstrating that AxyW harboring either L13Q (CIP102236-El9) or A37V (CIP102236-Eo4) has no repressive activity on AxySUV.

In silico modeling indicated that the substitutions L13Q and A37V in AxyW are located in the helix 1 and in the helix 3 of the N-terminal DNA-binding domain of AxyW, respectively (Figure S5).

It is noteworthy that AxySUV is not present in all *A. xylosoxidans* isolates. In Genbank, we identified 168 complete genomes of *A. xylosoxidans*, including 109 with listed STs on pubMLST. A total of 47 different STs were identified among the 109 available genomes. The presence or absence of the efflux system was constant in strains belonging to the same ST. Among the 47 STs, when selecting one genome per ST, only 25/47 (53%) harbored the AxySUV efflux system. Phylogenetic analysis including one genome per ST showed that the corresponding strains were not clustered, regardless of the approach (Figure S6). The system, when present, is highly conserved among the strains (>98% nucleotidic similarity for the entire sequence from *axyW* to *axyV*) and located between genes encoding a protein named XoxI (gene number 2, Figure S7) and a hypothetical protein (gene number 6, Figure S7). These last two genes are also present, but contiguous, in isolates not harboring the efflux system, for instance, in R4 (accession number: LN890476). No mutation leading to L13Q or A37V amino acid substitutions in AxyW has been detected in the available genomes harboring AxySUV. Other mutations leading to amino acid substitutions in AxyW have been detected in genomes available on Genbank. However, the absence of data concerning MICs or efflux system expression in these strains severely limits interpretation.

## 3. Discussion

This work has identified two previously undescribed RND-type efflux systems in clinically important *Achromobacter* species: AinCDJ in *A. insuavis* and AxySUV in *A. xylosoxidans*. Their implication in acquired resistance was demonstrated in one-step mutants selected in vitro on FQ.

The first system in *A. insuavis* was named AinCDJ because of common properties with MexCD-OprJ in *P. aeruginosa*. Besides sequence similarities, the overproduction of both systems leads to increased resistance to the same antibiotics (ofloxacin, ciprofloxacin, cefepime, meropenem, doripenem, minocycline, doxycycline, tigecycline, and chloramphenicol), while imipenem and ceftazidime are spared by these two effluxes [16,17,18]. Moreover, as described for MexCD-OprJ [19], the inactivation of AinCDJ does not modify the wild-type susceptibility of the parental strain, strongly suggesting that this pump does not contribute to *A. insuavis* intrinsic antibiotic resistance.

The second efflux system from *A. xylosoxidans* was named AxySUV because it harbors fewer similarities with MexCD-OprJ than AinCDJ from *A. insuavis*. The systems AxySUV and AinCDJ also share limited similarities and are different RND efflux systems. First, limited amino acid similarities were observed between AxySUV and AinCDJ. Second, the genes encoding the AxySUV system are inconsistently detected in the *A. xylosoxidans* genomes available on Genbank, in contrast to those of the AinCDJ system in *A. insuavis*. Moreover, although constant within the same species, the genomic context of *axySUV* in *A. xylosoxidans* is different from that of *ainCDJ* in *A. insuavis*. Both systems are not involved in resistance to the antibiotics tested in our study unless overexpressed, leading to an increase in the MICs of FQ, doxycycline, minocycline, doripenem, and chloramphenicol. However, in contrast to AinCDJ and MexCD-OprJ, the overproduction of AxySUV is not associated with the increase in MICs of cefepime, tigecycline, and meropenem, suggesting different substrate profiles. Interestingly, in the CIP102236-Eo4 mutant overexpressing AxySUV, the MIC of cefepime was reduced, suggesting the involvement of another unidentified mechanism.

The activity of three efflux pump inhibitors (EPIs) (CCCP, PaβN, and 1-(1-Naphthylmethyl)-piperazine (NMP)) was studied, but the restoration of antibiotic activity was mainly poor for both AxySUV and AxyCDJ, overproducing mutants, and sometimes, we even noticed an increase in MICs. As already reported previously, an MIC decrease in the presence of EPIs can be strain-dependent [20]. In the description of AxyABM in *Achromobacter*, Bador did not notice a significant decrease in the MICs of antibiotic substrates in the presence of EPIs [7,10].

In this work, the involvement of the AxySUV and AinCDJ systems in resistance was hypothesized after the identification of non-silent mutations in *axyW* or *ainK*. These genes encode regulators belonging to the TetR family transcriptional regulators (TFTRs). The TetR family of transcriptional regulators is a large family of single-component signal transduction proteins described as being involved in various cellular functions, including the regulation of efflux. Several RND-type efflux systems have a local TetR-type regulator. For example, Nfxb negatively regulates MexCD-OprJ in *P. aeruginosa,* or AxyZ negatively regulates AxyXY-OprZ in *A. xylosoxidans* [12,18,21]. For AxyW and AinK, the results of this study also support a repressive activity on the expression of the genes encoding the AxySUV and AinCDJ systems, respectively. TFTRs are essentially organized into nine alpha helices and function as dimers. They are characterized by a variable C-terminal ligand-binding domain and an N-terminal DNA-binding domain (helices 1 to 3) harboring a highly conserved helix-turn-helix (HTH) motif (helices 2 and 3) [22,23]. Helix 3 is called the recognition helix because it is inserted into the major groove when it binds to DNA. The literature reports substitutions in TFTRs associated with acquired antibiotic resistance through overexpression of RND efflux systems, for example, the substitutions A38V in NfxB, a regulator of MexCD-OprJ in *P. aeruginosa,* or V29G in AxyZ, a regulator of AxyXY-OprZ in *A. xylosoxidans* [12,24]. Interestingly, the substitutions V29G in AxyZ, A38V in Nfxb, L14Q in AinK, and A37V or L13Q in AxyW are all situated in the N-terminal DNA-binding domain. More specifically, L13Q, L14Q, and V29G are located at the first helix, while A37V and A38V are located on helix 3, the DNA recognition helix. These observations support the hypothesis that these substitutions have an impact on its inhibitory activity. Furthermore, protein sequence alignments show that the A38V substitution in Nfxb in *P. aeruginosa* corresponds to the A37V substitution in AxyW in *A. xylosoxidans*. The L13Q substitution in AxyW in *A. xylosoxidans* corresponds to the L14Q substitution in AinK in *A. insuavis*. These conserved residues appear to be key elements in the protein’s function.

Notably, the overproduction of the AinCDJ or AxySUV systems compromises the activity of antibiotics, including FQ and cyclines, but also some carbapenems, which are widely used to treat severe infections or patients with CF (i.e., cefepime, meropenem, doripenem, FQ, minocycline, doxycycline, tigecycline, and chloramphenicol for AinCDJ in *A. insuavis* and meropenem, FQ, minocycline, doxycycline, and chloramphenicol for AxySUV in A. *xylosoxidans*). Chemical substances (other than FQ) involved in the regulation of expression of these systems remain to be explored. It is noteworthy that FQs are substrates of all RND efflux systems described to date in *Achromobacter* (AxyABM, AxyXY-OprZ, AxyEF-OprN, AxySUV, and AinCDJ).

## 4. Materials and Methods

### 4.1. Bacterial Strains and MIC Determination

Two clinical wild-type reference strains, *A. insuavis* AXX-A (CIP110540) and *A. xylosoxidans* CIP102236, and their corresponding one-step in vitro mutants, AXX-A-Do1, CIP102236-Eo4, and CIP102236-El9, were included in the study (Table 3 and MICs in Table 1 and Table 2). One-step in vitro mutants had been previously selected on FQs in a previous work [6]. The CIP102236 and AXX-A parental strains were plated (inoculum of 10^7^ bacteria) on Mueller–Hinton (MH) agar supplemented with ofloxacin or levofloxacin (with concentrations ranging from 0.25 to 32 mg/L). One-step in vitro mutants growing on the plates were selected after 48 h incubation: AXX-A-Do1 and CIP102236-Eo4 on ofloxacin (8 mg/L) and CIP102236-El9 on levofloxacin (4 mg/L).

MICs of 18 antibiotics were determined by the test strip method as already used in our former descriptions of the efflux system AxyXY-OprZ and AxyABM (ceftazidime, cefotaxime, cefepime, ertapenem, imipenem, meropenem, doripenem, aztreonam, ofloxacin, levofloxacin, ciprofloxacin, doxycycline, minocycline, tigecycline, cotrimoxazole, trimethoprime, sulfamethoxazole, and chloramphenicol). MICs were measured twice and repeated in the event of discordance.

### 4.2. WGS

WGS was performed as previously described to compare AXX-A-Do1, CIP102236-Eo4, and CIP102236-El9 with their parental isolate, AXX-A, or CIP102236, respectively [6]. The bacterial DNA of AXX-A, AXX-A-Do1, CIP102236, CIP102236-Eo4, and CIP102236-El9 was extracted with the Gentra Puregene Yeast/Bact kit (Qiagen, Germantown, MD, USA). DNA libraries were prepared using the Nextera XT DNA Library Preparation Kit (Illumina, San Diego, CA, USA) and sequenced with the MiSeq reagent V2 (300-cycles) kit (Illumina) on the MiSeq system, generating 150 bp long paired-end reads. Draft genomes were assembled, annotated, and compared using the BV-BRC v3.49.1 software (https://www.bv-brc.org/, accessed on 12 March 2025) with default parameters (the annotation was performed by specifying the species: *A. xylosoxidans* or *A. insuavis*). For each genome, it was checked that the average depth was greater than or equal to 100 and the completeness score was greater than 95%.

Mutations found in the genes encoding the putative regulators, *axyW* and *ainK*, were verified by Sanger sequencing with the primers listed in Table S2.

### 4.3. Relative Gene Expression Measurement of ainD and axyU

The transcript levels of genes encoding the putative transporters (*ainD* or *axyU*) were compared between the mutants, the recombinants, and their parental strains by using RT-qPCR as previously described [6,12]. Briefly, total RNA was extracted using the RNeasy minikit (Qiagen), and reverse transcription was performed with the ImProm-II reverse transcription system (Promega, Madison, WI, USA) according to the manufacturer’s instructions. For qPCR, primers were designed for this study for the two targeted genes, *axyU* and *ainD,* and the housekeeping gene *rpoD* was used for the normalization (Table S2). For each gene, the relative expression was determined using the method described by Pfaffl [25], and the mean value was calculated from six independent measurements (two technical replicates from three distinct RNA extractions).

### 4.4. Gene Inactivation

The inactivation of genes encoding putative transporters or regulators (*axyU* or *axyW* in CIP102236, CIP102236-Eo4, CIP102236-El9; *ainD* or *ainK* in AXX-A and AXX-A-Do1) was performed by homologous recombination using a suicide plasmid, as previously described [6,8]. To this end, specific primers were designed to amplify, respectively, a region located within the gene (*axyU*, *axyW*, *ainD*, or *ainK*) (Table S2).

The cloning of each PCR product was performed in a pUC19 vector using the HD cloning kit (Clontech Laboratories, Mountain View, CA, USA) and following the manufacturer’s recommendations. The newly constructed plasmids (p-INA-axyU, p-INA-axyW_CIP102236_, p-INA-axyW_Eo4_, p-INA-axyW_El9_, p-INA-ainD, and p-INA-ainK) (Table S1) were introduced in strains by electroporation. Recombinant clones (named CIP102236-ΔU, CIP102236-ΔW, CIP102236-Eo4-ΔU, CIP102236-Eo4-ΔW, CIP102236-El9-ΔU, CIP102236-El9-ΔW, AXX-A-ΔD, AXX-A-ΔK, AXX-A-Do1-ΔD, and AXX-A-Do1-ΔK) were selected on MH agar plates containing 50 mg/L of ticarcillin (marker of pUC19). The disruption, i.e., the incorporation of the plasmid into the target gene (*axyU*, *axyW*, *ainD,* or *ainK*) in strains was checked by PCR and DNA sequencing using specific primers (Table S2).

### 4.5. In Silico Detection of axySUV and ainCDJ in Available Genomes and Phylogenetic Analysis

After the determination of the species for all *Achromobacter* fully sequenced genomes available in Genbank through pubMLST (https://pubmlst.org/, accessed on 27 March 2025), we selected all isolates belonging to *A. xylosoxidans* or *A. insuavis* to search for the prevalence of AxySUV or AinCDJ.

Sequences of *A. xylosoxidans* were used to determine the sequence type (ST) and to obtain phylogenetic trees. A first approach was carried out using the “Bacterial Genome Tree” tool on the BV-BRC platform to generate a tree using 1000 randomly selected genes and default parameters [26]. We also used a second approach by aligning the concatenated MLST sequences (2249 bp) [3] for each ST with the Simple Phylogeny tool of the ClustalW2 package (https://www.ebi.ac.uk/jdispatcher/phylogeny/simple_phylogeny, accessed on 27 March 2025) using the default parameters. The iTOL V6 online tool (https://itol.embl.de/, accessed on 31 March 2025) was used to format the tree data obtained (Figure S6).

### 4.6. Nucleotide Sequence Accession Numbers

The nucleotide sequence of *axyW*, *axyS*, *axyU,* and *axyV* in CIP102236 were assigned GenBank accession numbers: OP186062, OP186063, OP186064, and OP186065, respectively.

## 5. Conclusions

We described two novel efflux systems in *Achromobacter*: AxySUV in *A. xylosoxidans* and AinCDJ in *A. insuavis*. The overproduction of these systems might contribute to acquired antibiotic resistance. The impact of these mechanisms in clinical strains remains to be evaluated.

## Figures and Tables

**Table 1:** MICs of 18 antibiotics for reference clinical strain AXX-A, its mutant AXX-A-Do1 mutant overexpressing the AinCDJ system, and their recombinants inactivated in target genes.

Antibiotic	MIC (mg/L)					
	AXX-A	AXX-A-ΔD	AXX-A-ΔK	AXX-A-Do1	AXX-A-Do1-ΔD	AXX-A-Do1-ΔK
Ofloxacin	4	2	≥32 (↑≥8)	≥32 (↑≥8)	2 (↓≥16)	≥32
Levofloxacin	1	1	16 (↑16)	8 (↑8)	1 (↓8)	16
Ciprofloxacin	0.5	1	16 (↑32)	8 (↑16)	1 (↓8)	16
Ceftazidime	4	4	4	4	4	4
Cefotaxime	≥32	≥32	≥32	≥32	≥32	≥32
Cefepime	32	32	128 (↑4)	≥256 (↑≥8)	32 (↓≥8)	≥256
Ertapenem	0.06	0.06	0.06	0.06	0.06	0.06
Imipenem	2	2	2	2	2	2
Meropenem	0.25	0.25	0.5	0.5	0.125 (↓4)	0.5
Doripenem	0.5	0.5	2 (↑4)	2 (↑4)	0.5 (↓4)	2
Aztreonam	≥256	≥256	≥256	≥256	≥256	≥256
Doxycycline	4	4	16 (↑4)	16 (↑4)	4 (↓4)	16 (↑4)
Minocycline	4	4	16 (↑4)	16 (↑4)	4 (↓4)	16 (↑4)
Tigecycline	2	2	8 (↑4)	8 (↑4)	2 (↓4)	8 (↑4)
Cotrimoxazole	0.03	0.03	0.03	0.03	0.06	0.03
Trimethoprim	≥32	≥32	≥32	≥32	≥32	≥32
Sulfamethoxazole	0.5	0.5	0.5	0.5	0.5	0.5
Chloramphenicol	32	32	≥256 (↑≥8)	≥256 (↑≥8)	32 (↓≥8)	≥256

Values in brackets: factors of MIC variation compared with the parental strains (↓, n-fold decrease; ↑, n-fold increase). Only factors >2 were considered relevant. (AXX-A-Do1, AXX-A-∆D, and AXX-A-∆K compared to AXX-A; AXX-A-Do1-∆D and AXX-A-Do1-∆K compared to AXX-A-Do1.)

**Table 2:** MICs of 18 antibiotics for reference clinical strain CIP102236, its mutants CIP102236-Eo4 and CIP102236-El9 overexpressing the AxySUV system, and their recombinants.

Antibiotic	MIC (mg/L)								
	CIP102236	CIP102236-ΔU	CIP102236-ΔW	CIP102236-Eo4	CIP102236-Eo4-ΔU	CIP102236-Eo4-ΔW	CIP102236-El9	CIP102236-El9-ΔU	CIP102236-El9-ΔW
Ofloxacin	4	8	≥32 (↑≥8)	≥32 (↑≥8)	8 (↓≥4)	≥32	≥32 (↑≥8)	4 (↓≥8)	≥32
Levofloxacin	2	2	8 (↑4)	16 (↑8)	4 (↓4)	16	16 (↑8)	2 (↓8)	16
Ciprofloxacin	2	4	≥32 (↑≥16)	32 (↑16)	4 (↓8)	16	32 (↑16)	2 (↓16)	≥32
Ceftazidime	2	2	1	1	2	1	2	2	1
Cefotaxime	≥32	≥32	≥32	≥32	≥32	≥32	≥32	≥32	≥32
Cefepime	64	64	16 (↓4)	16 (↓4)	64 (↑4)	16	64	64	16 (↓4)
Ertapenem	0.06	0.03	0.03	0.03	0.06	0.03	0.03	0.06	0.03
Imipenem	4	2	2	2	4	2	2	2	2
Meropenem	0.25	0.125	0.25	0.25	0.25	0.25	0.25	0.125	0.25
Doripenem	0.5	0.5	2 (↑4)	2 (↑4)	0.5 (↓4)	2	2 (↑4)	0.5 (↓4)	2
Aztreonam	≥256	≥256	128 (↓≥2)	128 (↓≥2)	≥256 (↑≥2)	128	≥256	≥256	128 (↓≥2)
Doxycycline	8	8	32 (↑4)	32 (↑4)	8 (↓4)	32 (↑4)	32 (↑4)	8 (↓4)	32 (↑4)
Minocycline	4	4	16 (↑4)	16 (↑4)	4 (↓4)	16 (↑4)	16 (↑4)	4 (↓4)	16 (↑4)
Tigecycline	2	2	4 (↑2)	4 (↑2)	4	4	4 (↑2)	2 (↓2)	4
Cotrimoxazole	0.03	0.03	0.03	0.03	0.06	0.03	0.03	0.06	0.03
Trimethoprim	≥32	≥32	≥32	≥32	≥32	≥32	≥32	≥32	≥32
Sulfamethoxazole	0.5	0.5	0.5	0.5	0.5	0.5	0.5	0.5	0.5
Chloramphenicol	64	32	64	128	32 (↓4)	64	≥256 (↑≥4)	64 (↓≥4)	64 (↓≥4)

Values in brackets: factors of MIC variation compared with the parental strains (↓, n-fold decrease; ↑, n-fold increase). Only factors >2 were considered relevant. (CIP102236-Eo4, CIP102236-El9, CIP102236-∆U, and CIP102236-∆W compared to CIP102236; CIP102236-Eo4-∆U and CIP102236-Eo4-∆W compared to CIP102236-Eo4; CIP102236-El9-∆U and CIP102236-El9-∆W compared to CIP102236-El9.)

**Table 3:** *Achromobacter* strains used in this study.

Strain Name	Description	Source or Reference
*Achromobacter insuavis*		
AXX-A (CIP110540)	Parental strain, wild type	Our collection
AXX-A-ΔD	AXX-A with *ainD* inactivated	This study
AXX-A-ΔK	AXX-A with *ainK* inactivated	This study
AXX-A-Do1	in vitro one-step mutant of AXX-A previously selected on ofloxacin (8 mg/L), *ainU* overexpressed (79-fold)	This study
AXX-A-Do1-ΔD	AXX-A-Do1 with *ainD* inactivated	This study
AXX-A-Do1-ΔK	AXX-A-Do1 with *ainK* inactivated	This study
*Achromobacter xylosoxidans*		
CIP102236	Parental strain, wild type	Institut Pasteur collection
CIP102236-ΔU	CIP102236 with *axyU* inactivated	This study
CIP102236-ΔW	CIP102236 with *axyW* inactivated	This study
CIP102236-Eo4	in vitro one-step mutant of CIP102236 previously selected on ofloxacin (8 mg/L), *axyU* overexpressed (138-fold)	This study
CIP102236-Eo4-ΔU	CIP102236-Eo4 with *axyU* inactivated	This study
CIP102236-Eo4-ΔW	CIP102236-Eo4 with *axyW* inactivated	This study
CIP102236-El9	in vitro one-step mutant of CIP102236 previously selected on levofloxacin (4 mg/L), *axyU* overexpressed (37-fold)	This study
CIP102236-El9-ΔU	CIP102236-El9 with *axyU* inactivated	This study
CIP102236-El9-ΔW	CIP102236-El9 with *axyW* inactivated	This study

## Data Availability

The original contributions presented in this study are included in the article/Supplementary Material. Further inquiries can be directed to the corresponding author.

## Supplementary Materials

The following supporting information can be downloaded at https://www.mdpi.com/article/10.3390/antibiotics14060536/s1. Table S1. *Escherichia coli* strain and plasmids used in this study. Table S2. Primers used in this work. Figure S1. Protein alignment of regulators: AxyW, AinK, and Nfxb. Figure S2. Alignment of membrane fusion proteins: AxyS, AinC, and MexC. Figure S3. Alignment of RND transporters: AxyU, AinD, and MexD. Figure S4. Alignment of outer membrane factors: AxyV, AinJ, and OprJ. Figure S5. In silico modeling of TetR-family transcriptional regulator and N-terminal DNA-binding domain of AinK, AxyZ, AxyW, and Nfxb using the online tool Swiss-model. Figure S6. Phylogenetic trees using 48 *A. xylosoxidans* genome sequences available in Genbank and belonging to different STs and distribution of the presence of *axySUV* within the different STs. Figure S7. Genomic context of AxyW and AxySUV in *Achromobacter xylosoxidans* strains. Reference [27] is cited in the supplementary materials.
