# Supplementary material for: Description of Two Resistance-Nodulation-Cell Division Efflux Systems Involved in Acquired Antibiotic Resistance: AxySUV in Achromobacter xylosoxidans and AinCDJ in Achromobacter insuavis
Source: Antibiotics (Basel). 2025 May 23;14(6):536. doi: 10.3390/antibiotics14060536 (PMC12189693; doi:10.3390/antibiotics14060536)
Supplement: Supplementary file 1 [file antibiotics-14-00536-s001.zip › antibiotics-3624078-supplemenatary.pdf]

Table S1. *Escherichia coli* strain and plasmids used in this study

	Description	Source or reference
Escherichia coli strain		
HST08 (Stellar^{TM})	For recombinant DNA manipulations	Clontech Takara Bio Group
Plasmids		
pUC19	Used as suicide vector in *Achromobacter*	Clontech Takara Bio Group
p-INA-ainD	Suicide vector pUC19 used for *ainD* inactivation in AXX-A and AXX-A-Do1	This study
p-INA-ainK	Suicide vector pUC19 used for *ainK* inactivation in AXX-A and AXX-A-Do1	This study
p-INA-axyU	Suicide vector pUC19 used for *axyU* inactivation in CIP102236, CIP102236-Eo4 and CIP102236-El9	This study
p-INA-axyW$_{CIP102236}$	Suicide vector pUC19 used for *axyW* inactivation in CIP102236	This study
p-INA-axyW$_{Eo4}$	Suicide vector pUC19 used for *axyW* inactivation in CIP102236-Eo4	This study
p-INA-axyW$_{El9}$	Suicide vector pUC19 used for *axyW* inactivation in CIP102236-El9	This study

Table S2. Primers used in this work

		Nucleotide sequence (5'-3')	Expected size (bp)
**Primers used for Sanger sequencing**			
ainK	*ainK-insuavis*-F1	CGGGTCGATGCGGAACAG	1220
	ainK-insuavis-R1	CGGCTACAACGATCCCAACT	
axyW	*axyW-xylo*-F1	AGGAGGGCAAGCAGGCATAA	916
	axyW-xylo-R1	AAGGATACGCTGGCCTCGAT	
**Primers used for RT-qPCR**			
ainD	*ainD-insuavis*-qPCR-F2	CAACGACGTGTACTTCAAGGT	95
	ainD-insuavis-qPCR-R2	CGCGCAGGCTCTTGGCGAA	
axyU	*axyU-xylo*-qPCR-F1	ATCAATGTGCGGGGGCAGTT	114
	axyU-xylo-qPCR-R1	TCCCATTTCGAGACGGGCAA	
rpoD	rpoD-qPCR-F1	AAGGACGGCTACAAGTCGGA	101
	rpoD-qPCR-R1	GCCAGCTTCTCGACCATCTT	
**Primers used for gene inactivation**			
ainD	INA-*ainD*-AXXH2-F	*cggtacccggggatc*GACGTGCAGAACCGCATCAAG	952
	INA-*ainD*-AXXH2-R	*cgactctagaggatc*CGATGGCGTCGTCCACCAG	
	V-INA-*ainD*-AXXH2-F	CTTCGAGCCCGGCACCGAT	
	V-INA-*ainD*-AXXH2-R	GGCCATGATGCGTTCGACGT	
ainK	INA-*A.ins-ainK*-F1	*cggtacccggggatc*ACCCTGCAGGAACTGGCCAA	323
	INA-*A.ins-ainK*-R1	*cgactctagaggatc*AGCGTTTCCTGGTAGCCGGA	
	V-INA-*A.ins-ainK*-F1	ACGCCAGGGTTGATGCTTGA	
	V-INA-*A.ins-ainK*-R1	AATCCGGTGGTTACCAGCGT	
axyU	INA-*xylo-axyU*-F1	*cggtacccggggatc*TACACGCTGATCCCGACCAT	578
	INA-*xylo-axyU*-R1	*cgactctagaggatc*AGCACGGCATACACCAGCAT	
	V-INA-*xylo-axyU*-F1	AACGCCGATGGTTCCACCGT	
	V-INA-*xylo-axyU*-R1	TCCAGTGTGCGGGACAAGGT	
axyW	INA-*xylo-axyW*-F1	*cggtacccggggatc*TCTGACGGTCTGGACGACAA	560
	INA-*xylo-axyW*-R1	*cgactctagaggatc*AGCTTCTGCACCATCGAGGT	
	V-INA-*xylo-axyW*-F1	AGATGCCGCCTCAGGTCTGA	
	V-INA-*xylo-axyW*-R1	ACGCGCGCAGCTACACCTAT	
	M14F	CCAGGGTTTTCCCAGTCACGA	
	M14R	GCGGATAACAATTTCACACAGGA	

In lower italics : 15-bp extensions (5') that are complementary to the ends of the linearized vector.

```
AxyW-CIP102236      -MSDGLDDKLARALALSVAQQPRANLQQLARSAGISKATLYRIAPTRDAVIDLLLERATQ         59
AinK-AXX-A          MSEDAGHERLLIALAQAMVDQPRATLQELAKAVGVSKATLYRFCQTRDQLVTRLMTHSAQ         60
Nfxb-PAO1           MTLISHDERLIKALAVAIVDRPRATLKELAEAAGVSKATLHRFCGTRDNLVQMLEDHGET         60
                        . .::*  *** ::.::***.*::**.:.*:*****:*:. *** ::  *  :.

AxyW-CIP102236      YLQDALVRAELETPPYAAALQRLTEAVVQGREFYMFWNHAQWVRVIDSRTVDLSVPIPSF         119
AinK-AXX-A          VMKQTLADSRLDAAPTREALRNLIEQHLAHKELTVFLMY-NWKPDMEMQ--ADIMQSWSG         117
Nfxb-PAO1           VLNQIIQACDLEHAEPLEALQRLIKEHLTHRELLVFLVF-QYRPDFLDP--HGEGARWQS         117
                     ::: :  . *:      **:.* :  :  :*: :*  . ::   :           .

AxyW-CIP102236      YGQALENFFLNGQKAGVFRIDVPSLWLVRAYDFLLYAAIDAAQRGEIAPLGMTSMVQKLF         179
AinK-AXX-A          YQETLDAFFLRGQREGVFRVDITAAALTELLITVITSMVDAERRGRIARLGIATVAEQML         177
Nfxb-PAO1           YLEALDAFFLRGQQKGVFRIDITAAVFTELFITLVYGMVDAERRGRAASSNSAHTLEQMF         177
                    * ::*: ***.**: ****:*: :  :..    :: . :** :**. *  . :   ::::

AxyW-CIP102236      LEGASDARA---      188
AinK-AXX-A          LHGISAEPAAAA      189
Nfxb-PAO1           LHGASNPARS--      187
                    *.* *
```

Figure S1. Protein alignment of regulators: AxyW, AinK and Nfxb. Alignment carried out with the online tool «CLUSTAL O(1.2.4) multiple sequence alignment » (https://www.ebi.ac.uk/jdispatcher/msa/clustalo). For each protein name, the strain name is also indicated: CIP102236 (*A. xylosoxidans*), AXX-A (*A. insuavis*) and PAO1 (*P. aeruginosa*). Genbank accession numbers: OP186062 for AxyW, EGP44028 for AinK and NP_253290 for Nfxb. Meaning of the following symbols (https://ebi-biows.gitdocs.ebi.ac.uk/documentation/faqs/clustal/): An * (asterisk) indicates positions which have a single, fully conserved residue. A : (colon) indicates conservation between groups of strongly similar properties. A . (period) indicates conservation between groups of weakly similar properties.

```
AxyS-CIP102236      MSQFTTS--GAALCLLALLAGCGGQEPQA-PQEQTVEVAPLVVAPRAQAIMADLPGRISP         57
AinC-AXX-A          -------MAVLAAGLVAGLAGCGGDNAPT-AAAEPRPVQVLAVQPQRYALASTLPGRVEP         52
MexC-PAO1           MADLRAIGRIGALAMAIALAGCGPAEERQEAAEMVLPVEVLTVQAEPLALSSELPGRIEP         60
                               *  :   *****  :           *  *.*  .  *: : ****:.*

AxyS-CIP102236      VRVAEVRARVAGIVRKRHFEEGSTVKEGDLLFTIEPAPFEAALARAQGALARAEAQVRQA         117
AinC-AXX-A          VRVAEVRARVAGIVLSREFEEGADVKAGDVLFRIDPAPFKAALSRAEGDLAKADAALSDA         112
MexC-PAO1           VRVAEVRARVAGIVVRKRFEEGADVKAGDLLFQIDPAPLKAAVSRAEGELARNRAVLFEA         120
                    **************  :.****: ** **:** *:***::**::**:* **:  * : :*

AxyS-CIP102236      QALVDRYAPLVRIEAVSRQEYDDAAAALQTAKANRVSAQAEVKTAQLDLGYASVRAPISG         177
AinC-AXX-A          QAVVRRYTPLVKIEAVSQQDFDTATAALKSAQAARRSAQADVETARLNLDYATVKAPISG         172
MexC-PAO1           QARVRRYEPLVKIQAVSQQDFDTATADLRSAEAATRSAQADLETARLNLGYASVTAPISG         180
                    ** * ** ***:*:***:*::* *:* *::*:*   ****:::**:*:*.**:* *****

AxyS-CIP102236      RIGRGLVTEGSLVGQGESTPMALIQQIDPVYADFRQPINAVLKLREAAEAGHVRAGGALE         237
AinC-AXX-A          RIGRAQVTEGALVGQNEATVMAKIQQLDPIYVDFTQPVADMLRMRTAMQTGELGQEE--G         230
MexC-PAO1           RIGRALVTEGALVGQGEATLMARIQQLDPIYADFTQTAAEALRLRDALKKGTLAAGD--S         238
                    ****. ****:****.*:* ** ***:**:*.** *     *::* * : * :

AxyS-CIP102236      PAVGLRVDGTGYTARGRLLFSDVSVDPGTGQVLLRAEFPNPDGLLLPGMYVRVSAEQGVD         297
AinC-AXX-A          AAISISIDGTDRTRSGRLLFSDIAVDRGTGQVSLRGEFANPDVLLLPGMYVRVQTRQGVD         290
MexC-PAO1           QALTLRVEGTPYERQGALQFADVAVDRGTGQIALRGKFANPDGVLLPGMYVRVRTPQGID         298
                     *: : ::**     * * *:*::** ****: **.:* *** :********* : **:*

AxyS-CIP102236      RQAIFVPQRAVMRGPDGQARVLVVSAQGRAQERAVQTGAMQGAQWQVTQGLAAGDQVVVD         357
AinC-AXX-A          PDAILVPQRAVVRSTDGKPQVLLVGQDDVVETRAVRTGTMRGADWHIVEGLAAGDRVIVG         350
MexC-PAO1           NQAILVPQRAVHRSSDGSAQVMVVGADERAESRSVGTGVMQGSRWQITEGLEPGDRVIVG         358
                     :**:****** *. **. :*::*. :  .: *:* **.*:*: *::.:**  **:*:*.

AxyS-CIP102236      GAAKIAPGTPLHVKQPDAQAPAHPPSR------        384
AinC-AXX-A          GVNAAVPGQKVSVTAAPPAD-KVAAAPDAVSRQ        382
MexC-PAO1           GLAAVQPGVKIVPKPDGAQAQAQSPAPQQ----        387
                    *     **  :  .           :
```

Figure S2. Alignment of membrane fusion proteins: AxyS, AinC and MexC. Alignment carried out with the online tool «CLUSTAL O(1.2.4) multiple sequence alignment » (https://www.ebi.ac.uk/jdispatcher/msa/clustalo). For each protein name, the strain name is also indicated: CIP102236 (*A. xylosoxidans*), AXX-A (*A. insuavis*) and PAO1 (*P. aeruginosa*). Genbank accession numbers: OP186063 for AxyS, EGP44029 for AinC and NP_253289 for MexC. Meaning of the following symbols (https://ebi-biows.gitdocs.ebi.ac.uk/documentation/faqs/clustal/): An * (asterisk) indicates positions which have a single, fully conserved residue. A : (colon) indicates conservation between groups of strongly similar properties. A . (period) indicates conservation between groups of weakly similar properties.

```
AxyU-CIP102236      MPNFFIQRPNFALVIAIFIALAGLLAISSLPVAQYPSVAPPQVIVRAVYPGASAGTINDS      60
AinD-AXX-A          MSRFFIDRPKFAWVVALFISLAGLLAIPSLPVAQFPVVAPPQVTINAVYPGASASTLVDS      60
MexD-PAO1           MSEFFIKRPNFAWVVALFISLAGLLVISKLPVAQYPNVAPPQITITATYPGASAKVLVDS      60
                    * .***.**:** *:*:**:*****.* .*****:* *****: : *.****** .: **

AxyU-CIP102236      VTSLIEEELNGAKGLLYYESQSNGSGVAEITVTFEPGTDPDLAQVDVQNRIKRVESRLPQ      120
AinD-AXX-A          VTSVIEEELNGAKHLLYYESSSSSSGSAEITVTFEPGTDPALAQVDVQNRIKKAEARLPT      120
MexD-PAO1           VTSVLEESLNGAKGLLYFESTNNSNGTAEIVVTFEPGTDPDLAQVDVQNRLKKAEARMPQ      120
                    ***::**.***** ***:** ....* ***.********* *********:*:.*:*:*

AxyU-CIP102236      SVMKEGLQLEQASSSFLLIYALTYKEE--GKDQVGLADYAARNINNEIRRVPGVGRVQMF      178
AinD-AXX-A          AVTQQGLQVEQASSNFLMIYALTYKGDDSGKDVVGLSDYAARHVNNEIRRIDGVGKVQFF      180
MexD-PAO1           AVLTQGLQVEQTSAGFLLIYALSYKEGAQRSDTTALGDYAARNINNELRRLPGVGKLQFF      180
                    :*  :***:**:*:.**:****:**     .* ..*.*****::***:**: ***::*:*

AxyU-CIP102236      AAERALRIWIDPAKLVGYGLSVDDVNRAIAAQNVQVSGGATGDQPSPSSQEITATINVRG      238
AinD-AXX-A          GAEAAMRVWIDPQKLLGVGLSVADVNAAIAAQNVQVPAGSFGSRPGSPEQELTATLAVKG      240
MexD-PAO1           SSEAAMRVWIDPQKLVGFGLSIDDVSNAIRGQNVQVPAGAFGSAPGSSAQELTATLAVKG      240
                    .:* *:*:**** **:* ***: **. ** .***** .*: *. *.   **:***: *:*

AxyU-CIP102236      QFSTIEEFAGIVLRANADGSTVRLGDVARLEMGRQDYRMGSRLNGQPAAAMGVQLAPGAN      298
AinD-AXX-A          TLDTPEEFGRIVLRANADGSSVRLADVARMEVGRQSYDFETRLNGRKAVGAAVQLAPGAN      300
MexD-PAO1           TLDDPQEFGQVVLRANEDGSLVRLADVARLELGKESYNISSRLNGTPTVGGAIQLSPGAN      300
                     :.  :**. :***** *** ***.****:*:*::.* : :****  :.. .:**:****

AxyU-CIP102236      ALETAKGIKARLAELSGNFPSDISYSVPFDTSVFVDVAIKKVVMTLAEAVVLVFLVMLLF      358
AinD-AXX-A          AINTVKAVKQRLQELSASFPEDVAYSVPFDTSRFVSVAIEKVVHTLIEAMVLVFLVMFLF      360
MexD-PAO1           AIQTATLVKQRLAELSAFFPEDMQYSVPYDTSRFVDVAIEKVIHTLIEAMVLVFLVMFLF      360
                    *::*.. :* ** ***. **.*: ****:*** **.***:**: ** **:*******:**

AxyU-CIP102236      LQNFRYTLIPTIVVPICLLGTLAVMLPLGFSVNMMTMFGMVLAIGILVDDAIVVVENVER      418
AinD-AXX-A          LQNVRYTLIPAIVVPVCLLGTFAVMSVLGFSVNMMTMFGMVLAIGILVDDAIVVVENVER      420
MexD-PAO1           LQNVRYTLIPSIVVPVCLLGTLMVMYLLGFSVNMMTMFGMVLAIGILVDDAIVVVENVER      420
                    ***.******:****:*****: ** *********************************

AxyU-CIP102236      LMAEEGLSPRDATVKAMGQISGAIVGITLVLAAVFLPLAFMTGSVGVIYRQFSVSLAVSI      478
AinD-AXX-A          IMAEEGLSPREATIKAMGQVSGAIIGITMVLAAVFLPLAFMSGSVGVIYRQFSLSLAVSI      480
MexD-PAO1           IMAEEGISPAEATVKAMKQVSGAIVGITLVLSAVFLPLAFMAGSVGVIYQQFSVSLAVSI      480
                    :*****:** :**:*** *:****:***:**:*********:*******:***:******

AxyU-CIP102236      LFSGFLALTLTPALSVLLLKPVAQGHHE-KKGFFGWFNRVFARMTERYTATTARLLARTG      537
AinD-AXX-A          LFSGFLALTFTPALCATFLKPIPKGHHEEKRGFFGAFNRFFTRLTSRFESLNSRLVRRTG      540
MexD-PAO1           LFSGFLALTFTPALCATLLKPIPEGHHE-KRGFFGAFNRGFARVTERYSLLNSKLVARAG      539
                    *********:****.. :***: :**** *:**** ***.*:*:*.*:   .::*: *:*

AxyU-CIP102236      RVMLVYAVLVGALGYAYTVLPSAFLPTEDQGYMNTDVQLPPGATLSRTLETTRQLEQYLG      597
AinD-AXX-A          RYMVIYAAIVAMLGVLYTRLPESFVPPEDQGYVIVDMQLPPGATHLRTDKVVADVEQHLM      600
MexD-PAO1           RFMLVYAGLVAMLGYFYLRLPEAFVPAEDLGYMVVDVQLPPGASRVRTDATGEELERFLK      599
                    * *::** :*. **  *  **.:*:* ** **: .*:******:  **  .  ::*:.*

AxyU-CIP102236      TRPAVADVLALQGFSFSGQGQNAGLGFVMFKDWARRAGSESAMAEADRANQALAGVPDGV      657
AinD-AXX-A          SLDAMGDAFTVMGFSFSGTGQNAGIAFPTLKDWSERGEGQSANDVADSVNGRFAVIDDGT      660
MexD-PAO1           SREAVASVFLISGFSFSGQGDNAALAFPTFKDWSERGAEQSAAAEIAALNEHFALPDDGT      659
                    :  *:...: : ****** *:**.:.*  :***:.*.  :**        *  :*   **.

AxyU-CIP102236      LFSAVPPPVEGMGNATGFSLRLQDRAGLGREALLAATETLIRKVYEAPQTFSYIMVEGLS      717
AinD-AXX-A          VMAVNPPPIEGLGNSGGFALRLQDRGGVGRAALTAARDELLAKANSSP-VIAYAMMEGLA      719
MexD-PAO1           VMAVSPPPINGLGNSGGFALRLMDRSGVGREALLQARDTLLGEIQTNP-KFLYAMMEGLA      718
                    :::. ***::*:**: **:*** **.*:** **  *  : *: :    *  : * *:***:

AxyU-CIP102236      DAPELDVRIDRDKAEALGVPFDAINSALSTAFGSALVNEFPNHGRMQRVIVQAEPESRAT      777
AinD-AXX-A          DAPQLRLDIDRQKAEALGVGFDVINTAISSAYGSATVNDFANAGRLQRVVVQADVRDRMT      779
MexD-PAO1           EAPQLRLLIDREKARALGVSFETISGTLSAAFGSEVINDFTNAGRQQRVVIQAEQGNRMT      778
                    :**:* : ***:**.**** *:.*. ::*:*:**  :*:* * ** ***::**:  .* *

AxyU-CIP102236      PESVARLNVMNRSGELVPLESFSEIGWKHGPVQLIRYNGYPSIKLNGDAAYGSSTGQAMK      837
AinD-AXX-A          PESVLKLNVPNKSGGMVPLAAFVETRWETGPVQVSRYNGYPAFKIAGDARPGHSTGEAMA      839
MexD-PAO1           PESVLELYVPNAAGNLVPLSAFVSVKWEEGPVQLVRYNGYPSIRIVGDAAPGFSTGEAMA      838
                    **** .* * * :* :*** :* .  *: ****: ******::::: ***  * ***:**

AxyU-CIP102236      EIERLVGQLPHGIGFEWTGLSYQEKAAGSQAPMLLALALLVVFLVLVALYESWKIPASVL      897
AinD-AXX-A          EIERIASELPAGIGYQWTGLSLQEKFAGSQAPMLFALAFLVVFLLMVALYESWAIPASVM      899
MexD-PAO1           EMERLASQLPAGIGYEWTGLSYQEKVSAGQATSLFALAILVVFLLLVALYESWSIPLSVM      898
                    *:**:..:** ***::***** *** :..**  *:***:*****::******* ** **:

AxyU-CIP102236      LIVPVGALGAVAAVIVAGMPNDVYFKVGLVTIIGLAAKNAILIIEFAKDLHAQGRTLREA      957
AinD-AXX-A          LIVPIGALGSVLAVTVLGMPNDVYFKVGLITIIGLAAKNAILIVEFAKSLREQGHSLVDA      959
MexD-PAO1           LIVPIGAIGAVLAVMVSGMSNDVYFKVGLITIIGLSAKNAILIVEFAKELWEQGHSLRDA      958
                    ****:**:*:* ** * ** *********:*****:*******:****.*  **::* :*

AxyU-CIP102236      AIEAARLRFRPIVMTSVAFILGVVPLVIATGAGATSQRAIGTGVLGGMLSATILGVLFVP      1017
AinD-AXX-A          AVEAAKLRFRPIVMTSLAFILGVVPLALASGAGGASQRALGVGVIGGMLSATLLGVIFVP      1019
MexD-PAO1           AIEAARLRFRPIIMTSMAFILGVIPLALASGAGAASQRAIGTGVIGGMLSATFLGVLFVP      1018
                    *:***:******:***:******:**.:*:***.:****:*.**:*******:***:***

AxyU-CIP102236      VFFVWTLSLLERRKTQAASRPLAPIQEGEK 1047
AinD-AXX-A          VFFVWVLSRIKARKAATPTPAAATASQE-- 1047
MexD-PAO1           ICFVWLLSLLRSKPAPIEQAASAGE----- 1043
                    : *** ** :. : :       *
```

Figure S3. Alignment of RND transporters: AxyU, AinD and MexD. Alignment carried out with the online tool «CLUSTAL O(1.2.4) multiple sequence alignment » (https://www.ebi.ac.uk/jdispatcher/msa/clustalo). For each protein name, the strain name is also indicated: CIP102236 (*A. xylosoxidans*), AXX-A (*A. insuavis*) and PAO1 (*P. aeruginosa*). Genbank accession numbers: OP186064 for AxyU, EGP44030 for AinD and NP_253288 for MexD. Meaning of the following symbols (https://ebi-biows.gitdocs.ebi.ac.uk/documentation/faqs/clustal/): An * (asterisk) indicates positions which have a single, fully conserved residue. A : (colon) indicates conservation between groups of strongly similar properties. A . (period) indicates conservation between groups of weakly similar properties.

```
OprJ-PAO1           --------MRKPAFGVSALLIALTLGACSMAPTYERPAAPVADSWSGAAAQ----RQGAA         48
AxyV-CIP102236      MNRTLNRVLARLKPLAAAAVALALLSGCALAPTYQRPASPVAQHWDADGQAGAASAPGSA         60
AinJ-AXX-A          MNPTF------ISSRLAAAILAAALAGCSMAPTYQRPEAPVPSTWNQPAAG----GPASA         50
                                    :* :    *..*::****:** :** . *. .        .:*

OprJ-PAO1           IDTLDWKSFIVDAELRRLVDMALDNNRSLRQTLLDIEAARAQYRIQRADRVPGLNAAATG         108
AxyV-CIP102236      ASVLDWRDFVTDPSLRGLVETALANNRDLRRALLNVEAARAMYRVQRAERLPGIGAQASG         120
AinJ-AXX-A          AATLDWQSFVTDDGLRRLVTLALDHNRDLRQALLNIEAARAQYRVQRADRLPGINAQGSG         110
                      .***:.*:.*  ** **  ** :**.**::**::***** **:***:*:**:.* .:*

OprJ-PAO1           NRQRQPADLSAGNRSEVASSYQVGLALPEYELDLFGRVKSLTDAALQQYLASEEAARAAR         168
AxyV-CIP102236      SRQHNPADLNPAGSAGVQSEWRAGLGVNAFELDLFGRVRSLSEAALEEYLATEAGARGAR         180
AinJ-AXX-A          TRQRVPGDLNSSGSAGVQSNYQAGIGLTSFEIDLFGRVRSLSDAALQEYLATEATARGAQ         170
                    .**: *.**. .. : * *.::.*:.:  :*:******:**::***::***:*  **.*:

OprJ-PAO1           IALVAEVSQAYLSYDGALRRLALTRQTLVSREYSFALIDQRRAAGAATALDYQEALGLVE         228
AxyV-CIP102236      ISLIAEVIEAYLTRESARQRGLHTERTLASREASLQLIGQRRAEGIGSALDYEQAKGLAD         240
AinJ-AXX-A          ISLVAEVIQAYLARDSALRRLQVTRQTLESREASLDLTAKRRQAGSATALDYQEALGLAE         230
                    *:*:*** :***: :.* :*   *.:** *** *: *  :**  * .:****::* **.:

OprJ-PAO1           QARAEQERNLRQKQQAFNALVLLLGSDDAAQAIP-RSPGQRPKLLQDIAPGTPSELIERR         287
AxyV-CIP102236      QARADLARMEREYRQSTNALTLLAGTNDLSPYLAPHDDAAAPLLVQQLAAGVPSELLALR         300
AinJ-AXX-A          QARADLERIDREARQAGNALALLVGVGDLGPFLP-KGLADGPMLVQEIAAGAPSELLERR         289
                    ****:  *  *: :*: ***.** * .* .  :  :. .  * *:*::* *.****:  *

OprJ-PAO1           PDILAAEHRLRARNADIGAARAAFFPRISLTGSFGTSSAEMSGLFDGGSRSWSFLPTLTL         347
AxyV-CIP102236      PDILAAEHRLRARNADIGAARAAFFPRISLTGMFGSASADLSDLFKGGQRAWSFAPQLTL         360
AinJ-AXX-A          PDIVAAEHQLQSRNASIGAARAAFFPSISLTGMFGSSSAELSNLFDSGQRAWSFAPQITL         349
                    ***:****:*::***.********** ***** **::**::*.**..*.*:*** * :**

OprJ-PAO1           PIFDGGRNRANLSLAEARKDSAVAAYEGTIQTAFREVADALAASDTLRREEKALRALANS         407
AxyV-CIP102236      PLFDGGRNQANLDLAKLRKDMAVAEYEQSIQTAFREVMDGLAATDTLRRQEAAQQAQADS         420
AinJ-AXX-A          PIFAGGRNTANLDLANARKDIAVAQYEKTVQSAFREVSDALAATDTLRREEASRLALTQS         409
                    *:* **** ***.**: *** *** ** ::*:***** *.***:*****:* :  * ::*

OprJ-PAO1           SNEALKLAKARYESGVDNHLRYLDAQRSSFLNEIAFIDGSTQRQIALVDLFRALGGGWDE         467
AxyV-CIP102236      SQAALRLSEARYRGGVDSYLRYLDAQRSDFVNQIALIEVRTQRQVALATLFRALGGGWRG         480
AinJ-AXX-A          SAQAMKLSEARYRGGVDSHLRYLDAQRRAYADQLSYIEVATQRQAALATLFKALGGGWLP         469
                    *  *::*::***..***.:********  : :::: *:  **** **. **:******

OprJ-PAO1           GRSLVVHRGGRS-----------     479
AxyV-CIP102236      DADAGQ-----------------     486
AinJ-AXX-A          SAPAPVAPGADKGAAPAGGQARG     492
                    .
```

Figure S4. Alignment of outer membrane factors: AxyV, AinJ and OprJ. Alignment carried out with the online tool «CLUSTAL O(1.2.4) multiple sequence alignment » (https://www.ebi.ac.uk/jdispatcher/msa/clustalo). For each protein name, the strain name is also indicated: CIP102236 (*A. xylosoxidans*), AXX-A (*A. insuavis*) and PAO1 (*P. aeruginosa*). Genbank accession numbers: OP186062 for AxyV, EGP44028 for AinJ and NP_253290 for OprJ. Meaning of the following symbols (https://ebi-biows.gitdocs.ebi.ac.uk/documentation/faqs/clustal/): An * (asterisk) indicates positions which have a single, fully conserved residue. A : (colon) indicates conservation between groups of strongly similar properties. A . (period) indicates conservation between groups of weakly similar properties.

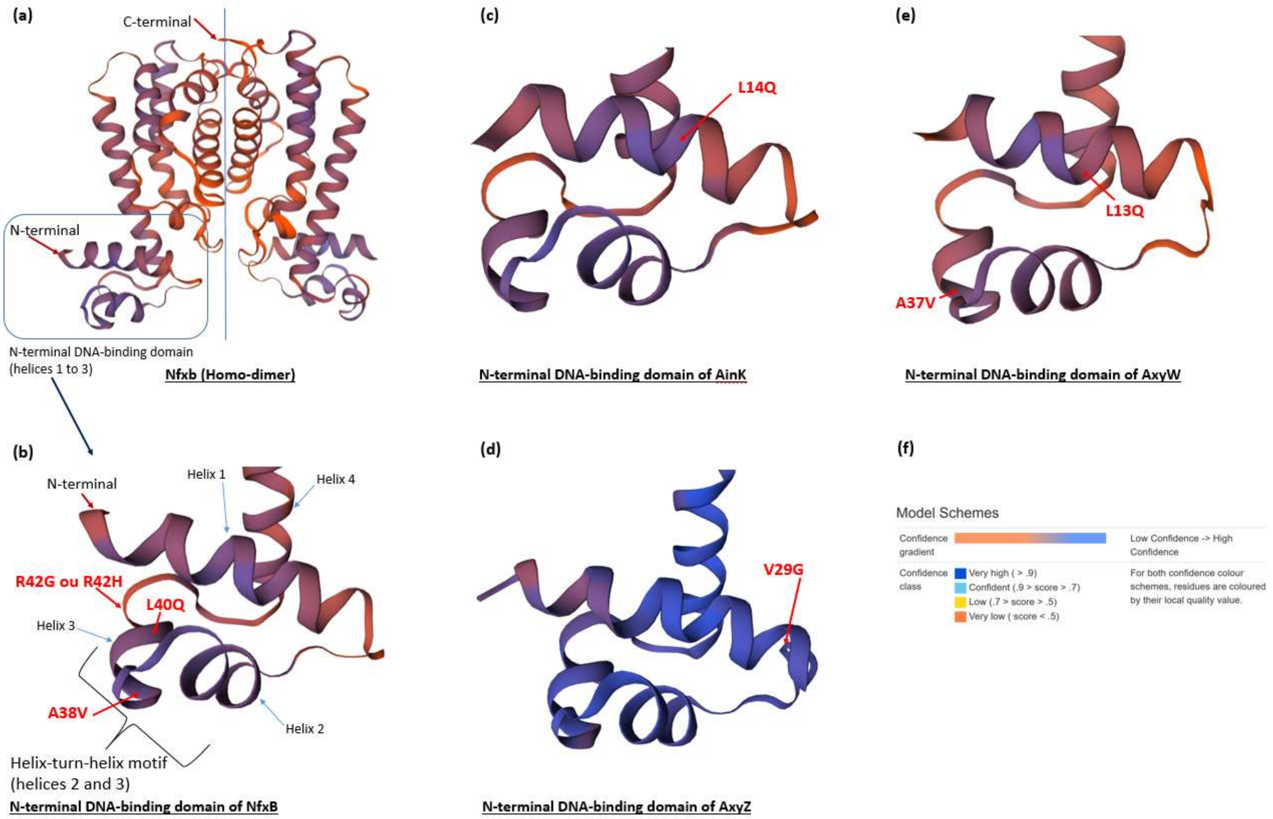

Figure S5. *In silico* modelling of Nfxb transcriptional regulator and N-terminal DNA-binding domain of Nfxb, AinK, AxyZ and AxyW using the online tool Swiss-model [27]. **(a)** Nfxb (PAO1 sequence) transcriptional regulator, repressor of the MexCD-OprJ system in *P. aeruginosa*, organisation in 9 alpha helices and a dimer (blue line representing the axis of symmetry), based on 5gpa.1.A template, « Structural analysis of fatty acid degradation regulator FadR from *Bacillus halodurans* » *Q9K8A4*. GMQE: 0,54 ; QMEANDisco Global: 0,58 +/- 0,05. **(b)** N-terminal DNA-binding domain of Nfxb and identification of the different helices and the helix-turn-helix motif. Helix 3 is described as the DNA recognition helix **(c)** N-terminal DNA-binding domain of AinK (AXX-A sequence), repressor of the AinCDJ system in *A. insuavis*, based on 5gpa.1.A template, « Structural analysis of fatty acid degradation regulator FadR from *Bacillus halodurans* » *Q9K8A4*. GMQE: 0,56 ; QMEANDisco Global: 0,61 +/- 0,05. **(d)** N-terminal DNA-binding domain of AxyZ (CIP102236 sequence), repressor of the AxyXY-OprZ system in *Achromobacter*, based on 2wui.1.A template, *«* Crystal structure of MexZ, a key repressor responsible for antibiotic resistance in *Pseudomonas aeruginosa »* *Q9RG61*. GMQE: 0,82 ; QMEANDisco Global: 0,79 +/- 0,05. **(e)** N-terminal DNA-binding domain of AxyW, repressor of the AxySUV system in *A. xylosoxidans* (CIP102236 sequence), based on 4w1u.1.A template, « Crystal structure of Rv3557c/KstR2, a transcriptional repressor involved in cholesterol metabolism in *Mycobacterium tuberculosis»* *P9WMB9*. GMQE: 0,53 ; QMEANDisco Global: 0,54 +/- 0,05. **(b-e)** The red arrows indicate the substitutions and their positions described as associated with overexpression of the corresponding efflux genes (This study and [12, 24]). **(f)** Colour code for structures from: https://swissmodel.expasy.org/docs/help#model_results.

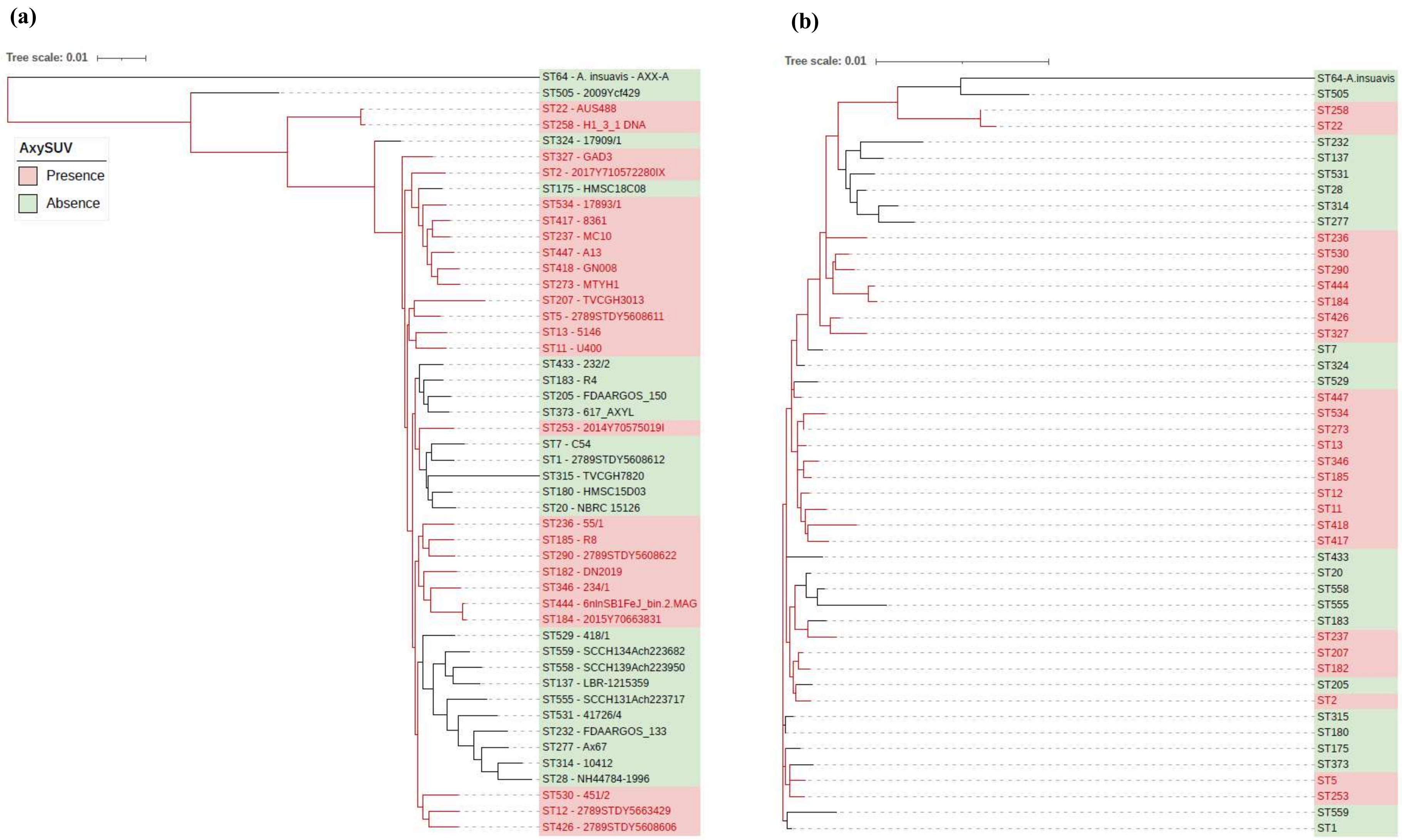

Figure S6. Phylogenetic trees using 48 *A. xylosoxidans* genome sequences available in Genbank and belonging to different STs and distribution of the presence of *axySUV* within the different STs. (**a**) Approach using the "Bacterial Genome Tree" tool on the BVBRC platform (1000 randomly selected genes and default parameters). (**b**) Approach based on the alignment of concatenated MLST sequences [1] using the Simple Phylogeny tool of the ClustalW2 package (default parameters).

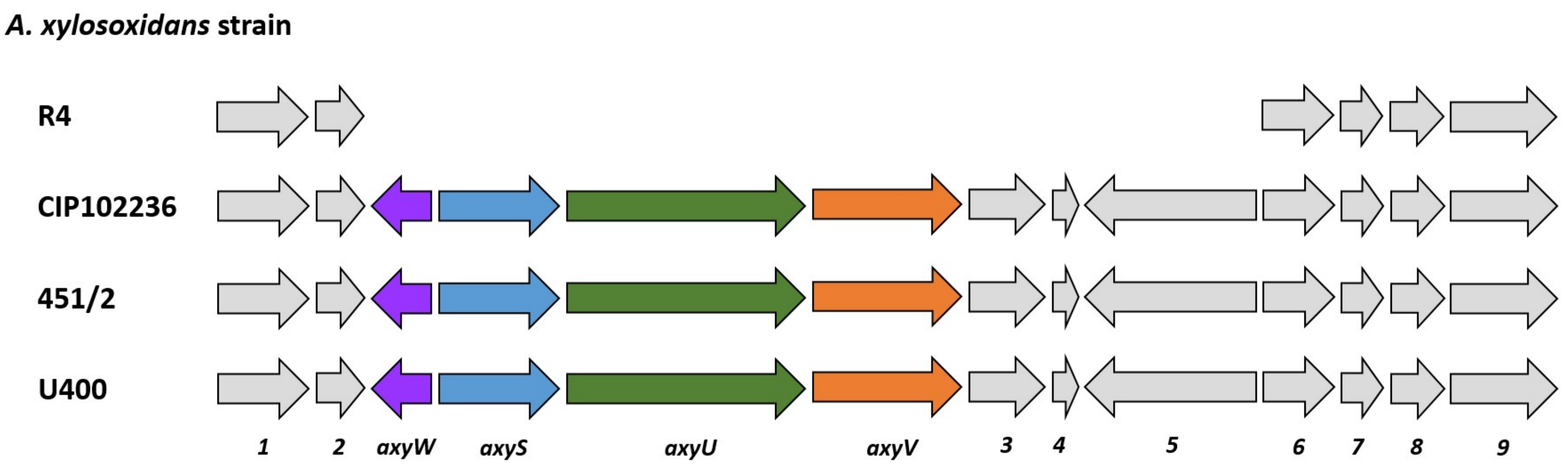

1 Uncharacterized dehydrogenase Pfl_3523
2 XoxI
3 Hypothetical protein
4 Hypothetical protein
5 Potassium efflux system KefA protein / Small-conductance mechanosensitive channel
6 Hypothetical protein
7 Transcriptional regulator, ArsR family
8 Arsenate reductase (EC 1.20.4.4) thioredoxin-coupled, LMWP family
9 Arsenical-resistance protein ACR3

Figure S7. Genomic context of AxyW and AxySUV in *Achromobacter xylosoxidans* strains. The genomic context of AxyW and AxySUV was analysed in 4 strains of *A. xylosoxidans*: R4 (accession number: LN890476), CIP102236, 451/2 (accession number: JAKJWR000000000) and U400 (accession number: MJMO00000000). The numbered genes correspond to the genes flanking those encoding AxyW and the AxySUV system. The proteins encoded by these genes are indicated in the legend and correspond to to those proposed by the genome annotation carried out with the BVBRC platform.
